# Patterns of Abnormal Glucose Metabolism in Acromegaly and Impact of Treatment Modalities on Glucose Metabolism

**DOI:** 10.7759/cureus.13852

**Published:** 2021-03-12

**Authors:** Sajjad Ali Khan, Nanik Ram, Muhammad Q Masood

**Affiliations:** 1 Department of Medicine, Section of Endocrinology, Aga Khan University Hospital, Karachi, PAK

**Keywords:** growth hormone and diabetes, acromegaly, diabetes mellitus, transsphenoidal surgery, somatostatin analogues, acromegaly and diabetes, acromegaly complications, acromegaly and surgery, acromegaly comorbidities

## Abstract

Background: Chronic exposure to high levels of growth hormone (GH) and insulin-like growth factor 1 (IGF-1) leads to metabolic complications, most importantly dysglycemia in the form of diabetes mellitus and pre-diabetes. Dysglycemia if diagnosed early in the course of the disease can decrease complications. Treatment modalities in the form of surgery and medical therapy have varied impacts on glucose metabolism.

Objective: To determine the frequency of diabetes mellitus, impaired glucose tolerance, and impaired fasting glucose in Pakistani patients with acromegaly and to establish the impact of the intervention (surgery/medical) on glucose metabolism.

Methods: This study was a retrospective review of patient records. Eighty-nine patients fulfilling the Endocrine Society criteria for acromegaly diagnosis were included. A data of baseline, GH, IGF-1 level, hemoglobin A1C (HbA1C), fasting blood glucose (FBG), and random blood glucose (RBS) levels were reviewed before and after the intervention (surgery/medical therapy). Normal glucose tolerance (NGT), impaired fasting glucose (IFG), impaired glucose tolerance (IGT), and diabetes mellitus (DM) were defined based on the American Diabetes Association (ADA) criteria. Patients were grouped into normoglycemic (NGT) and dysglycemic (IFG, IGT, and DM) based on FBG, RBS, and HbA1C.

Results: Major risk factors for dysglycemia included age (15-45 years), male sex (33.70%), obesity (45.7%), and macroadenoma (77.52%). Both mean GH levels (58.29 vs. 54.36 ng/dl) and IGF-1 levels (862.98 vs. 824.32 ng/dl) were higher among the normoglycemic than dysglycemia. Pre-surgery NGT, IFG, IGT, IFG, and IGT/DM combined were found in 48.31%, 5.61%, 1.1%, 5.61%, and 39.32% of the subjects respectively. Post-surgery, HbA1C improved in 79.5%, deteriorated in 6.8%, and remained the same in 13.6%. Similarly, it improved in 67% post-medical therapy. Both FBG and RBS improved post-surgery and medical therapy. Further, the number of anti-diabetic drugs used also decreased post-surgery.

Conclusion: Dysglycemia is more common among patients with acromegaly as compared to the general population and tends to be poorly controlled in untreated acromegaly. Glycemic control improves significantly after surgery and medical therapy.

## Introduction

Acromegaly is a rare disease entity caused by benign tumors of the pituitary gland for the most part that leads to hypersecretion of growth hormone (GH) and the resultant increase in insulin-like growth factor-1 (IGF-1) [1]. Chronic exposure to elevated GH and IGF-1 leads to several complications including cardiovascular and metabolic abnormalities, which lead to increased morbidity and mortality [2,3]. Among the metabolic complications, impaired metabolism of carbohydrates is well studied, with studies reporting a prevalence of 12-52.5% for overt diabetes mellitus (DM) [4-7], 14-26% for impaired glucose tolerance (IGT) and impaired fasting glucose (IFG) respectively [8].

Treatment options for acromegaly include surgical resection of the tumor and medical therapy to overcome the effects of GH and IGF-1. These modalities impact glucose metabolism in many ways [9]. Surgical resection of the tumor leads to a decrease in the levels of GH and IGF-1 and thus less insulin resistance in the body. It has been shown to positively impact glucose metabolism including fasting blood glucose (FBG), two-hour post-meal glucose, random blood glucose (RBS), and hemoglobin A1C (HbA1C) [9-11]. Among the drugs used to treat acromegaly, somatostatin analogs, especially pasireotide, have been shown to worsen glucose homeostasis leading to a deterioration of HbA1c, FBG, and random blood sugar levels [9], while bromocriptin is associated with improved glycemic tolerance [12]. It has also been shown that glucose metabolism is more related to the body mass index (BMI) rather than the surgical resection or somatostatin analogs [13].

This study aims to determine the prevalence of spectrum of impaired glucose metabolism including the IFG, IGT, and overt DM in a South Asian country, and to assess the impact of different treatment modalities on glucose metabolism in patients diagnosed with acromegaly presenting in a tertiary care hospital.

## Materials and methods

This is a retrospective review of patient records presented to the tertiary care hospital in Karachi, Pakistan, for the diagnosis and management of acromegaly. After the approval from the ethical review committee (ERC) of our center (approval 2020-4779-11081), we requested the health information and management services (HIMS) to provide the list of patients diagnosed to have pituitary tumors. 

A non-probability convenience sampling technique was used to recruit the study participants. A total of 89 patients fulfilled the diagnosis of acromegaly based on the clinical characteristics and biochemical markers and were included in the study from 2000 to 2020. These patients were either undergoing treatment with surgery/medical therapy for acromegaly or being followed up in a tertiary care hospital in Karachi, Pakistan.

The inclusion criteria included confirmed cases of acromegaly fulfilling the clinical features and endocrine society diagnostic criteria, i.e. 1) patients with elevated or equivocal serum IGF-1 levels matched for age and sex, and confirmation of the diagnosis by finding lack of suppression of GH to <1 μg/L following documented hyperglycemia during an oral glucose load [14].

With regards to dysglycemia, patients were classified according to the diagnostic criteria of the American Diabetes Association (ADA) [15]. Group 1 included patients with normal glucose tolerance (NGT) defined as FPG of less than 100 mg/dl, and/or a two-hour post 75-gram glucose load (OGTT) of less than 140 mg/dl and/or HbA1C of less than 5.7%.

Group 2 comprised patients with dysglycemia with either an impaired fasting glycemia between 100 and 125 mg/dl (IFG) or impaired glucose tolerance (IGT) with a two-hour post-OGTT-glucose between 140 and199 mg/dl and HbA1C between 5.7% to 6.5%. DM was defined as an FPG greater than 126 mg/dl or a two-hour post-OGTT-glucose 200 mg/dl or more and HbA1C greater than 6.5%.

Patients who did not come for follow-up post-surgery or medical therapy were excluded from the study. Patients aged 15 years or below were also excluded from the study, as body mass index (BMI) and blood pressure varied in childhood. Data were collected retrospectively by reviewing patient records for both baseline and subsequent visits post-intervention (surgery and medical therapy). For each patient, we analyzed the following data recorded by the primary physician at the diagnosis of acromegaly: age and sex, BMI, estimated duration since acromegaly diagnosis, mean serum GH, IGF-1 level (matched for age and sex in each center), pituitary tumor size measured by magnetic resonance imaging (MRI), and presence of FBG, two-hour glucose level (RBS) and HbA1C.

To assess the impact of surgery and medical therapy on glucose metabolism, patients were followed up with HbA1c, FBG, and RBS at three, six, and 12 months. Improvement and deterioration of diabetes control were assessed based on changes in the parameters of glucose monitoring i.e. FBG, RBS, and HbA1C.

Patients were stratified into the well-controlled group when HbA1C was less than 7%, fasting blood glucose (FBG) between 80- 130 mg/dl, and post-prandial or random blood sugar (RBS) between 140-200 mg/dl (a surrogate for two-hour post glucose).

HbA1C was considered acceptable or poorly controlled when it was between 7-8% or greater than 8% respectively. Similarly, FBG and RBS were considered uncontrolled when they exceeded 130 mg/dl and 200 mg/dl respectively.

Diabetes control was considered improved when the HbA1C level would decrease at least by 1% or FBG/RBS improve from the baseline readings. Similarly, diabetes was considered to deteriorate when HbA1C, FBG, and RBS would increase compared to baseline. Furthermore, anti-diabetic drugs used before and after the surgery were compared to see if surgery had any impact on the number of drugs being used.

Statistical analysis

Patient data were initially entered in Excel and then all statistical analyses were performed using Statistical Package for Social Sciences (SPSS) version 24.0 (IBM Corp., Armonk, NY, USA). A descriptive analysis was conducted to report the demographics characteristics of the study cohort. For qualitative variables, frequencies and percentages were reported for different variables. For continuous variables, mean and standard deviation (SD) were reported. The glycemic control was also reported according to the treatment modalities provided to the patients.

## Results

Table 1 illustrates the general characteristics of the study population. The majority of the study patients were young, falling in the age range of 15 to 45 years (77.54%) with 64% being males. Most of the patients had a BMI between 25-35 (77.51%) and were diagnosed within five years of disease onset (67.41%).

**Table 1:** General characteristics of patients. BMI, body mass index

		Total Patients (N)=89			
		Normoglycemics=43 (48.3%)		Dysglycemics=46 (51.68%)	
		N (%)		N (%)	
Age	15-30 years	17 (19.1)		9 (10.11)	
	30-45 years	23 (25.84)		20 (22.47)	
	>45 years	3 (3.37)		17 (19.10)	
Gender	Male	27 (30.33)		30 (33.70)	
	Female	16 (18)		16 (18)	
BMI	<25	9 (10.11)		3 (3.37)	
	25-30	16 (17.97)		17 (19.10)	
	30-35	15 (16.85)		21 (23.59)	
	>35	3 (3.37)		5 (5.61)	
Tumor size	Macroadenoma (>1 cm)	34 (38.20)		35 (39.32)	
Duration of disease (acromegaly) diagnosis	1-5 years	31 (34.83)		29 (32.58)	
	5-10 years	10 (11.23)		7 (7.86)	
	>10-15 years	2 (2.24)		10 (11.23)	

Our study shows that among acromegalics, the prevalence of NGT, DM, IFG, IGT, and combined IFG and IGT was 48.31%, 39.32%, 5.61%, 1.1%, and 5.61% respectively. Sixty-nine patients (77.5%) had macroadenoma. All of the 89 (100%) patients underwent transsphenoidal surgery (TSS) of the pituitary tumor and 25 patients (28%) received medical therapy in the form of somatostatin analog (SSA; octreotide LAR), cabergoline, bromocriptine, or a combination of the above for the residual disease post-TSS.

Table 2 illustrates that the mean levels of GH were higher in the normoglycemic group 58.29 ± 122.47 ng/ml as compared to the dysglycemic group 54.36 ± 74.32 ng/ml. Similarly, IGF-1 levels were higher in the normoglycemic patients than those with dysglycemia (862.98 ± 319.07 vs 824.32 ± 30.81 ng/ml).

**Table 2:** Growth hormone and insulin-like growth factor levels pre- and post-surgery

Laboratory Parameter	Pre-surgery		Post-surgery	
	Mean ± SD		Mean ± SD	
	Dysglycemics N=46	Normoglycemics N=43	Dysglycemics N=46	Normoglycemics N=43
Growth hormone (GH) (ng/ml)	54.36 ± 74.32	58.29 ± 122.47	40.80 ± 94.50	47.16 ± 204.39
Insulin-like growth factor( IGF-1) (ng/ml)	824.32 ± 30.81	862.98 ± 319.07	739.33 ± 368.42	646.33 ± 389.35

Table 3 illustrates that majority had uncontrolled glycemic control before the surgery which improved significantly post-surgery. The impact was evident from the improved levels of HbA1C, FBG, and RBS. Similarly, these parameters improved in patients receiving the medical therapy.

**Table 3:** Impact of treatment modalities (surgery and medical therapy) on glycemic control HbA1C, hemoglobin A1C; FBG, fasting blood glucose; RBS, random blood sugar

		Pre-Surgery Total Number=46 N (%)	Pre-Medical Total number=25 Therapy N (%)		Post-Surgery Total number=46 N (%)	Post-Medical Therapy Total Number=25 N (%)
HbA1C (%)	<7	14 (30.4)	10 (40)	Improved	35 (79.5)	16 (64.0)
	7-8	11 (23.9)	10 (40)	Worsened	3 (6.8)	
	>8	17 (37)	4 (16)	No Effect	6 (13.6)	9 (36.0)
	Not known	4 (8.7)	1 (4)			
FBG	Controlled	20 (43.5)	14 (56)	Improved	31 (67.4)	10 (40)
	Uncontrolled	26 (56.5)	11 (44)	Worsened	7 (15.2)	3 (12)
				No Effect	8 (17.4)	12 (48)
RBS	Controlled	21(45.7)	13 (52)	Improved	28(60.9)	15 (6)
	Uncontrolled	25(54.3)	12 (48)	Worsened	1(2.2)	
				No Effect	17(37)	10 (40)

The number of drugs used to control diabetes (oral hypoglycemic and insulin) also reduced post-surgery. Our study shows that before the surgery 30.4% of patients had controlled diabetes without any drugs, which increased to 37% post-surgery. Similarly, patients who had diabetes control with one to two drugs (52.2%) and more than three drugs (17.4%) changed to 47.8% and 15.2% respectively.

Table 4 illustrates the impact of various drugs used to control the disease on glycemic control. Somatostatin analogue (octreotide LAR), cabergoline, bromocriptine, and combination therapy was used in 52%, 20%, 24%, and 4% respectively. Overall HbA1c, FBG, and RBS improved in 64%, 40% and 61% of patients post medical therapy. HbA1c, FBG, and RBS remained unchanged in 36%, 48%, and 40% of patients respectively. However, FBG deteriorated in 12% of patients.

**Table 4:** Impact of specific medical therapy (somatostatin analogue [SSA], cabergoline, bromocriptine, and combination therapy) on glycemic control.

	Pre-medical therapy					Post-medical therapy				
		SSA	Cabergoline	Bromocriptine	Combination therapy		SSA	Cabergoline	Bromocriptine	Combination therapy
		N (%)	N (%)	N (%)	N (%)		N (%)	N (%)	N (%)	N (%)
HbA1c	Well controlled (<7)	4 (16.0%)	4 (16.0%)	2 (8.0%)	-	Improved	9 (36.0%)	2 (8.0%)	4 (16.0%)	1 (4.0%)
	Acceptable control (7-8)	6 (24.0%)	-	3 (12.0%)	1 (4.0%)	No effect	4 (16.0%)	3 (12.0%)	2 (8.0.0%)	-
	Poorly controlled (>8)	3 (12.0%)	1 (4%)	-	-					
	Not known	-	-	1 (4.0%)	-					
Fasting blood glucose	Well controlled	6 (24.0%)	2 (8.0%)	5 (20.0%)	1 (4.0%)	Improved	6 (24.0%)	2 (8.0%)	1 (4.0%)	1 (4.0%)
	Poorly controlled	7 (28.0%)	3 (12.0%)	1 (4.0%)	-	Deteriorated	2 (8.0%)	1 (4%)	-	-
						No effect	5 (20.0%)	2 (8.0%)	5 (20.0%)	-
Random blood glucose	Controlled	7 (28.0%)	2 (8.0%)	3 (12.0%)	1 (4.0%)	Improved	7 (28.0%)	4 (16.0%)	4 (16.0%)	-
	Poorly controlled	6 (24.0%)	2 (8.0%)	2 (8.0%)		No effect	6 (24.0%)	1 (4.0%)	2 (8.0%)	1 (4.0%)
	Control unknown	-	1 (4.0%)	1 (4.0%)						

## Discussion

Our study shows a high prevalence of diabetes (39.32%) among acromegalic patients as compared to the general population of this country (19.4%) as shown in the International Diabetes Federation Diabetes Atlas, 9th edition [16]. Furthermore, the prevalence of prediabetes (IFG, IGT) was 12.35% among the acromegalic patients. This higher prevalence of 39.32% is in parallel with over 50% of prevalence reported in the Russian population reported by Dreval et al. [17], 31.9% in the Mexican population [18], 28% in the Belgian population [8], 22.3% in the French population [7] and 19% in Slovak Republic population [19]. Our study also reveals that glycemic control improves post-surgery and medical therapy reflected in the form of improved HbA1C, FBG, and RBS compared to pre-surgery and medical therapy levels.

Previous studies have shown that insulin resistance in acromegalic patients is multifactorial, with age, greater duration of the disease, higher BMI, and higher IGF-1 levels being primary risk factors [7,8]. There have been conflicting reports regarding the duration of acromegaly, age, and gender concerning the prevalence of diabetes in different populations. A study done in Mexico revealed that old age and female gender were more likely to be associated with the development of diabetes in acromegaly than young age and male [18]. Another study from Russia also revealed a higher prevalence of diabetes in older patients who had a longer duration of acromegaly [17].

In contrast to these studies, our study reveals that 32.58% of the patients with dysglycemia were relatively young (15-45 years) as compared to 19.10% of patients who were older than 45 years of age. Moreover, the duration of diabetes and gender was not correlated well with the previous studies as the majority (32.59%) of acromegalic patients in the dysglycemia group had symptoms for less than five years. Similarly, 33.70% of the patients in the dysglycemia group were male, compared to only 17.97% of female patients in the dysglycemic group. Our study is consistent with other studies that higher BMI is associated more with the onset of abnormal glucose metabolism. Only 3.37% of the dysglycemia group had a BMI of less than 25.

Studies have demonstrated the impact of tumor size and levels of IGF-1 and GH on the degree of insulin resistance. Niculescu et al. revealed that IGF-1 level is more likely to correlate with insulin resistance than GH [20]. In contrast, the French acromegaly registry did not show any significant association between the level of IGF-1 and GH and the risk of diabetes [7]. In another study done by Moisés Mercado et al. in Mexico, GH levels and larger tumor size (macroadenoma) were more likely to be associated with the risk of diabetes [21].

Consistent with these studies, our study also reveals that 77.52% of dysglycemics had macroadenoma (>1 cm). However, the levels of GH and IGF-1 were not strongly correlated with dysglycemia as the mean GH levels were higher in normoglycemics as compared to dysglycemics (58.29 vs 54.36 ng/dl). IGF-1 levels were also higher in normoglycemics (862.98 vs 824.32 ng/dl). These inconsistencies may be related to the relatively increased prevalence of diabetes and glucose intolerance in our population.

The study is consistent with other studies that untreated acromegaly is associated with a high incidence of abnormal glucose metabolism [17,19]. Studies have revealed that glycemic control improves post-surgery. A study done in Milan, Italy, revealed that glucose homeostasis, i.e. fasting glucose, postprandial glucose, and HbA1c, improves with surgery [22]. Other studies done in China and Poland also revealed a positive effect of surgery on glucose homeostasis [10,11]. In the Chinese study, higher pre-operative fasting C-peptide levels were correlated with improved diabetic control post-operatively. Our study further strengthens the idea that surgery improves glycemic control as all the parameters improve post-surgery as shown in the results.

Studies have reported varied impact of medical therapies on glucose metabolism. Two recent meta-analyses assessing the impact of somatostatin analogues (SSAs) on glucose metabolism revealed a worsening of two-hour postprandial glucose and HbA1C while the FBG remained unchanged [23,24]. In these studies, the patients were given only medical therapy and the effects on glycemic control were assessed. However, in our study medical therapy was given to patients with residual disease who had previously undergone the surgery, HbA1C improved by 64.0% and remained unchanged in 36% of those patients who received medical therapy in the form of somatostatin analogue SSA (Octreotide LAR), cabergoline, bromocriptine, and combination therapy. Similarly, FBG and RBS improved in those patients who received medical therapy.

In this study, we tried to compare the number of anti-diabetic drugs used before and after the surgery. Our study shows that before the surgery number of drugs was higher than post-surgery. 

Our study has a few limitations. Our study is a retrospective assessment of patient records presented to our center. Secondly, the study population is relatively small compared to other studies. Having said that, our study also has strengths. This is the first study of its kind in our country in which we specifically reviewed the impact of both surgery and medical therapy on glycemic control. Most of the studies done previously have assessed the impact of either surgery or medical therapy. In contrast, our study took into account the impact of surgery followed by medical therapy.

## Conclusions

Our study shows that untreated acromegaly is associated with dysglycemia in the form of diabetes mellitus, impaired fasting glucose, and impaired glucose tolerance. The rate of diabetes mellitus is much higher than the general population.

Our study reveals that early intervention in the form of surgery and medical therapy to control acromegaly can translate into better glycemic control in this cohort of patients.
